# Epidemiological characteristics and influencing factors of hand, foot and mouth disease reinfection cases in Jiulongpo District, Chongqing, China, 2009–2023

**DOI:** 10.3389/fpubh.2025.1543450

**Published:** 2025-03-19

**Authors:** Huixian Zhou, Yuan Yao, Qianjin Long, Chunyan Deng

**Affiliations:** Center for Disease Control and Prevention of Jiulongpo District, Chongqing, China

**Keywords:** hand, foot and mouth disease, reinfection, epidemiological characteristics, spatial autocorrelation, influencing factors

## Abstract

**Objective:**

To analyze the epidemiological characteristics of Hand, Foot and Mouth Disease (HFMD) reinfection and its influencing factors in Jiulongpo District from 2009 to 2023 to provide targeted prevention and control recommendations for key factors.

**Methods:**

HFMD cases in Jiulongpo District of Chongqing were derived from the China Information System for Disease Control and Prevention from 2009 to 2023. Descriptive analysis was used to analyze the epidemiological characteristics of HFMD reinfection, spatial autocorrelation to analyze the regional clustering, and binary logistic regression to analyze the influencing factors.

**Results:**

From 2009 to 2023, 4,764 HFMD reinfection cases involving 2,436 individuals were reported in Jiulongpo District, with a reinfection rate of 5.48%. The interval between the two infections ranged from 26 to 3,863 days, and 71.51% of patients were reinfected within 2 years. There was a bimodal distribution in time (April–July and October–November). In the population, the reinfection rate was 5.87% in males and 4.93% in females, 3.97% in scattered children and 7.89% in kindergarten children, 8.61% in children >3 years old, and 4.68% in children ≤3 years old. There was a spatial positive correlation of HFMD reinfection in Jiulongpo District, with hot spots concentrated in the rural area and cold spots in the urban area. The multifactorial logistic regression analysis showed that reinfection risk was higher in non-epidemic years, male, rural areas, >3 years old, and kindergarten children (*p* < 0.05).

**Conclusion:**

Post-epidemic prevention and control measures should prioritize interventions to target reinfection, focusing on children in rural areas and kindergartens. Improve rural infrastructure and sanitation, raise disease awareness in kindergartens, train healthcare workers, and promote hygiene to reduce HFMD reinfection.

## 1 Introduction

Hand, Foot and Mouth Disease (HFMD) is an acute infectious disease caused by enteroviruses, which is characterized by fever, hand and foot rashes, and oral blisters and is transmitted through the digestive tract, the respiratory tract, and through close contacts (1, 2). The common causative pathogens are human enterovirus 71 (EV-A71) and coxsackievirus 16 (CV-A16) (3, 4). HFMD mainly affects children under the age of 5 years, with a higher incidence in temperate regions during summer and autumn (5). Most mild cases are self-limiting, but in severe cases, it can lead to neurological complications, pulmonary edema, and even death (6, 7).

In China, since HFMD was included in the management of category C statutory infectious diseases in 2008, the average annual number of reported cases has exceeded 1 million (8), ranking first among all statutory infectious diseases reported (9). The annual economic losses due to HFMD in Beijing, the capital city of China, range from US$7.03 million to US$13.31 million (10) and will only be higher in economically underdeveloped areas. Chongqing Municipality is one of the high-risk areas for HFMD in China (11), and its incidence rate is higher than the national incidence rate (12). The severe economic and social burdens caused by HFMD have become an important public health problem that cannot be ignored.

Vaccination effectively and cost-effectively controls disease transmission, promotes health equity, and reduces mortality (13). In the first half of 2016, the first inactivated EV-A71 vaccine was marketed in mainland China (11), and clinical trials demonstrated that it had an efficacy rate of 97.4% (14) but did not provide cross-protection against HFMD caused by non-EV-A71 enteroviruses. Meanwhile, observational studies have shown that EV-A71 vaccine-induced antibody responses, lasting >2 years, are not lifelong, and reinfection is still possible (15). There are many types and subtypes of enteroviruses that cause HFMD, and there is no cross-immunity between different types and subtypes. Although a certain degree of specific immunity can be obtained after infection, it is only against the same type of virus, and there is no immunity against other types of viruses that are susceptible to reinfection (16). Therefore, reinfection is challenging to avoid. From 2008 to 2015, 398,010 HFMD patients (a total of ≈820,000 episodes) in China were diagnosed with reinfection cases (17).

Several studies have reported reinfection of HFMD, with reported reinfection rates ranging from 1.93 to 11.92% (18–21). Factors associated with HFMD reinfection include demographic characteristics, socioeconomic factors, and climatic conditions. Although HFMD epidemics are known to have distinct seasonal peaks (22) and epidemic years (23), few studies have examined how annual epidemic intensity and seasonal transmission peaks affect reinfection. Our study innovatively included epidemic year and peak periods in the analysis, aiming to comprehensively capture the epidemiological characteristics of reinfection and its influencing factors and to provide targeted prevention and control recommendations for key factors.

## 2 Materials and methods

### 2.1 Study area

**Figure 1:**
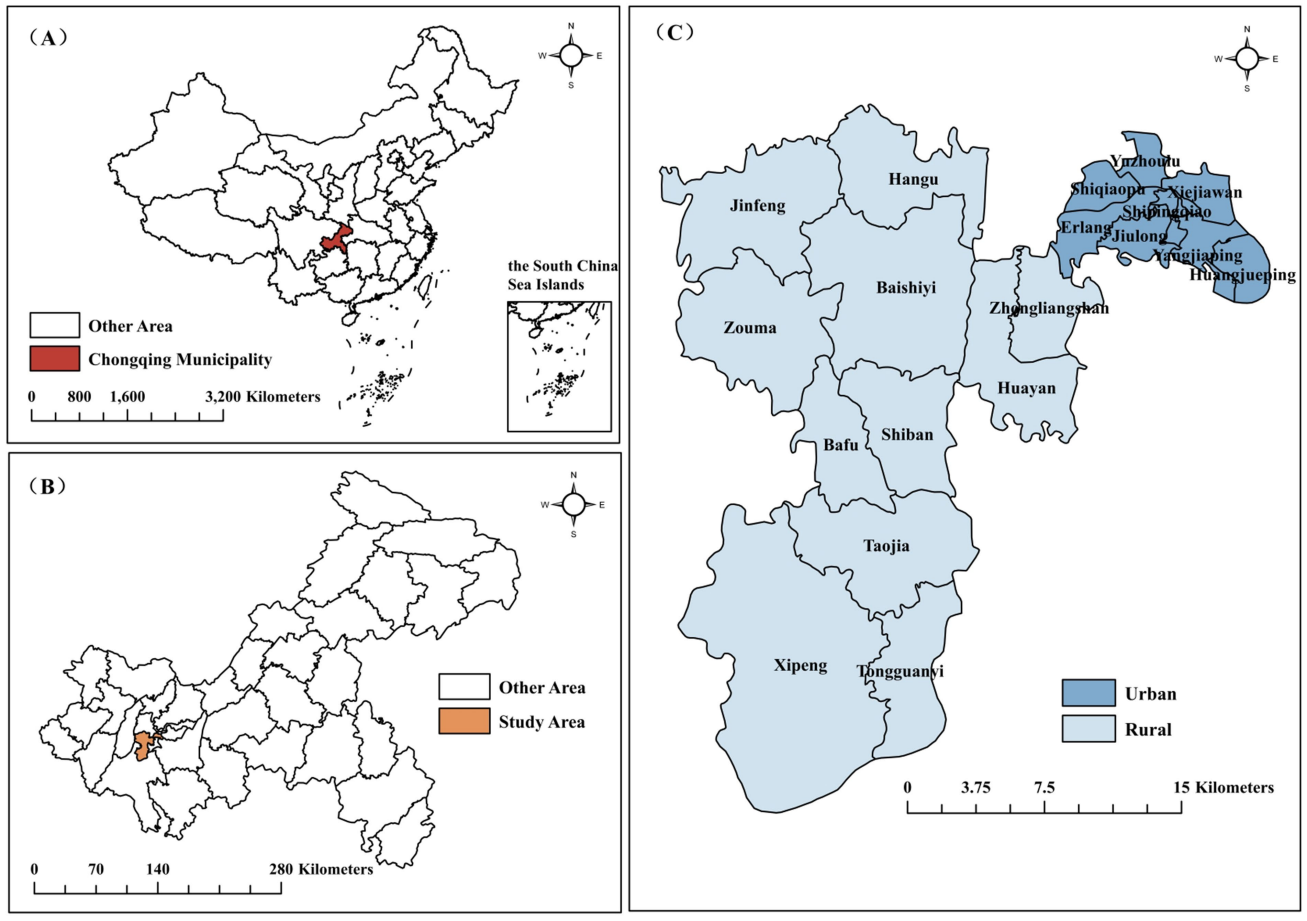
Geographical location of Jiulongpo District. **(A)** China; **(B)** Chongqing Municipality; **(C)** Jiulongpo District.

Chongqing is one of the four municipalities under the direct jurisdiction of the central government of China. Jiulongpo District is located in the Yangtze River Valley area, with a typical subtropical monsoon climate characterized by hot and dry summers, cloudy and rainy winters, humid air, and weak winds. It is favorable for the growth and reproduction of enterovirus, and the annual incidence rate of HFMD in Jiulongpo District is always in the top 2 positions in Chongqing. Jiulongpo District has a total area of 432 square kilometers, with 19 towns under its jurisdiction and a resident population of 1.54 million. The Zhongliang Mountain Range divides the Jiulongpo District into two. The urban areas east of the Zhongliang Mountains are relatively flat, economically developed, and highly urbanized. In contrast, the rural areas west of the Zhongliang Mountains are predominantly mountainous, economically poor, and poorly urbanized, as shown in Figure 1.

### 2.2 Data resources

The data for this study came from the China Information System for Disease Control and Prevention (CISDCP), an Internet-based surveillance system established by the Chinese government in 2004. Since May 2, 2008, healthcare facilities must report any suspected, clinically diagnosed, or laboratory-confirmed case of HFMD through CISDCP within 24 h. The basic information of the case includes the ID number, name, gender, age, occupation, address, date of onset, date of diagnosis, and case classification (Suspected cases, clinical cases, or laboratory-confirmed cases). The Center for Disease Control and Prevention in Jiulongpo District has taken a series of measures to ensure the quality of data reported on infectious diseases, such as training healthcare facilities on the standards for reporting infectious diseases on a biannual basis, checking outpatient and inpatient journals of healthcare facilities every month to make sure that there are no infectious diseases that have been incorrectly diagnosed or underreported, and auditing the report cards of reported infectious diseases daily, and so on. In this study, a database of 47,045 HFMD cases with onset dates between January 2009 and December 2023 and with a residential address in Jiulongpo District was exported from CISDCP.

### 2.3 Define

#### 2.3.1 HFMD cases

Diagnostic criteria for HFMD were based on *the diagnosis and treatment of hand, foot and mouth disease (2018 edition)* published by the National Health Commission of the People’s Republic of China. A clinical case of HFMD is a patient with a papular or blistering rash on the hands, feet, mouth, or buttocks, with or without fever. The infection can be laboratory-confirmed based on clinical diagnosis if it meets one of the following criteria: (1) detection of specific enterovirus nucleic acid sequences (CV-A16, EV-A71, etc); (2) Isolation and identification of enteroviruses such as CV-A16, EV-A71, or other type that could cause HFMD; (3) presence of IgM antibody against disease-related virus during the acute stage; (4) presence of neutralizing antibody titer against the relevant enterovirus in the recovery phase is at least four times higher than that in the acute stage.

#### 2.3.2 Reinfection

The same patient is considered to be reinfected if the previous infection was a mild case of HFMD and the interval between the onset of the subsequent infection was greater than 25 days, or a severe case of HFMD and the interval between the onset of the subsequent infection was greater than 60 days.

#### 2.3.3 Epidemic year

According to the results in Table 1, HFMD in Jiulongpo District showed high incidence in even-numbered years and low incidence in odd-numbered years from 2009 to 2020, so the even-numbered years between 2009 and 2020 were defined as epidemic years. Influenced by Coronavirus Disease, 2021–2023 showed high incidence in odd-numbered years and low incidence in even-numbered years, so the odd-numbered years between 2021 and 2023 were defined as epidemic years.

**Table 1:** Reinfections of HFMD in Jiulongpo District, 2009–2023.

Year	Non-reinfection	Reinfection					Reinfection rate (%)
		Individuals^a^	Cases^b^	2 Infections	3 Infections	4 Infections	
2009	1,026	1	2	1			0.10
2010	1,893	30	59	29	1		1.56
2011	1,404	54	108	54			3.70
2012	2,241	96	190	94	2		4.11
2013	2,254	90	176	86	4		3.84
2014	3,992	212	411	199	13		5.04
2015	2,971	223	439	216	6	1	6.98
2016	4,385	269	519	250	18	1	5.78
2017	2,236	202	396	194	7	1	8.29
2018	5,407	311	611	300	11		5.44
2019	3,592	385	751	366	18	1	9.68
2020	1,395	62	121	59	3		4.26
2021	3,081	187	362	175	11	1	5.72
2022	1,509	117	230	113	4		7.20
2023	4,646	197	389	192	5		4.07
Total	42,032	2,436	4,764	2,328	103	5	5.48

a Individuals means the number of persons with HFMD.

b Cases means the number of reports in CISDCP.

#### 2.3.4 Peak periods

According to the seasonal index in Table 2, the peak periods were April–July and October–November.

**Table 2:** Seasonality index and coefficients of variation of HFMD reinfection individuals in Jiulongpo District, 2009–2023.

Month	Reinfection	Seasonality index	Mean	Standard deviation	Coefficients of variation
1	101	0.53	8.42	6.26	0.74
2	46	0.32	5.11	2.47	0.48
3	98	0.47	7.54	5.58	0.74
4	255	1.23	19.62	15.05	0.77
5	364	1.76	28.00	23.69	0.85
6	475	2.30	36.54	29.33	0.80
7	267	1.12	17.80	15.59	0.88
8	57	0.26	4.07	4.16	1.02
9	70	0.37	5.83	4.45	0.76
10	217	1.05	16.69	13.63	0.82
11	302	1.35	21.57	13.37	0.62
12	184	0.96	15.33	9.52	0.62
Total	2,436	1.00	15.92	17.01	1.07

### 2.4 Inclusion and exclusion criteria

**Figure 2:**
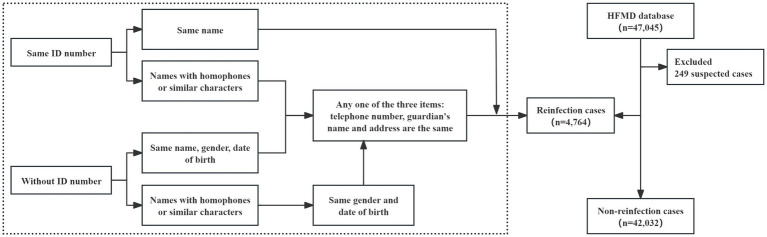
Reinfection case filtering process.

Inclusion criteria: (1) the address was Jiulongpo District, Chongqing; (2) the diagnosis was HFMD; (3) the onset time was between January 2009 and December 2023; (4) the case classification was clinical cases or laboratory-confirmed cases. Exclusion criteria: case classification was a suspected case. The filtering process is shown in Figure 2.

### 2.5 Analyses

The seasonal index (24) was used to examine the seasonal characteristics of HFMD reinfection with the following formula: seasonal index = Mean number of reinfected cases in the same month of the year / Mean number of reinfected cases in all months, with a seasonal index greater than 1 indicating a high prevalence of reinfection in that month. The coefficient of variation of the seasonal index also showed the seasonality of HFMD reinfection with the following formula: coefficient of variation = Standard deviation/Mean; the smaller the coefficient of variation, the more seasonal the month.

Spatial autocorrelation analysis was performed using ArcGIS 10.8 software. (1) Global spatial autocorrelation analysis selects Moran’s I index for determining whether there is a spatial aggregation of HFMD reinfection in Jiulongpo District, I > 0 means positive spatial correlation, and I < 0 means negative spatial correlation. (2) Local spatial autocorrelation analysis selected Getis-Ord Gi^*^ index to identify whether there were statistically significant hotspots and coldspots in the local area, Gi^*^>0 meant that the area was a high-value spatial aggregation (hotspots), and Gi^*^<0 meant that the area was a low-value spatial aggregation (coldspots).

Influence factor analysis was performed using R 4.4.1 software, where the chi-square test was used to compare differences in reinfection rates on factors such as gender, age, region, and population classification, and binary logistic regression was used for multifactorial analyses. Statistical significance was considered at *p* < 0.05.

## 3 Results

### 3.1 HFMD reinfection

From 2009 to 2023, 2,436 individuals (the number of persons with HFMD) /4,764 cases (the number of reports in CISDCP) were reported to be reinfected with HFMD in Jiulongpo District, with a reinfection rate of 5.48%. The top 3 years of reinfection rate were 2019 (9.68%), 2017 (8.29%), 2022 (7.20%), and other relatively low years with reinfection rates ranging from 0.10 to 6.98%. Five individuals were infected 4 times (0.21%), 103 individuals were infected 3 times (4.23%), and 2,328 individuals were infected twice (95.57%). There were 45 cases of severe illness in the first infections, of which 2 cases of reinfection occurred and manifested as mild illnesses, as shown in Table 1.

#### 3.1.1 Time distribution

According to the seasonal index, HFMD reinfection individuals showed two incidence peaks in 2009–2023: April–July and October–November. The coefficient of variation was smaller in November, suggesting a strong seasonality, as shown in Table 2.

**Figure 3:**
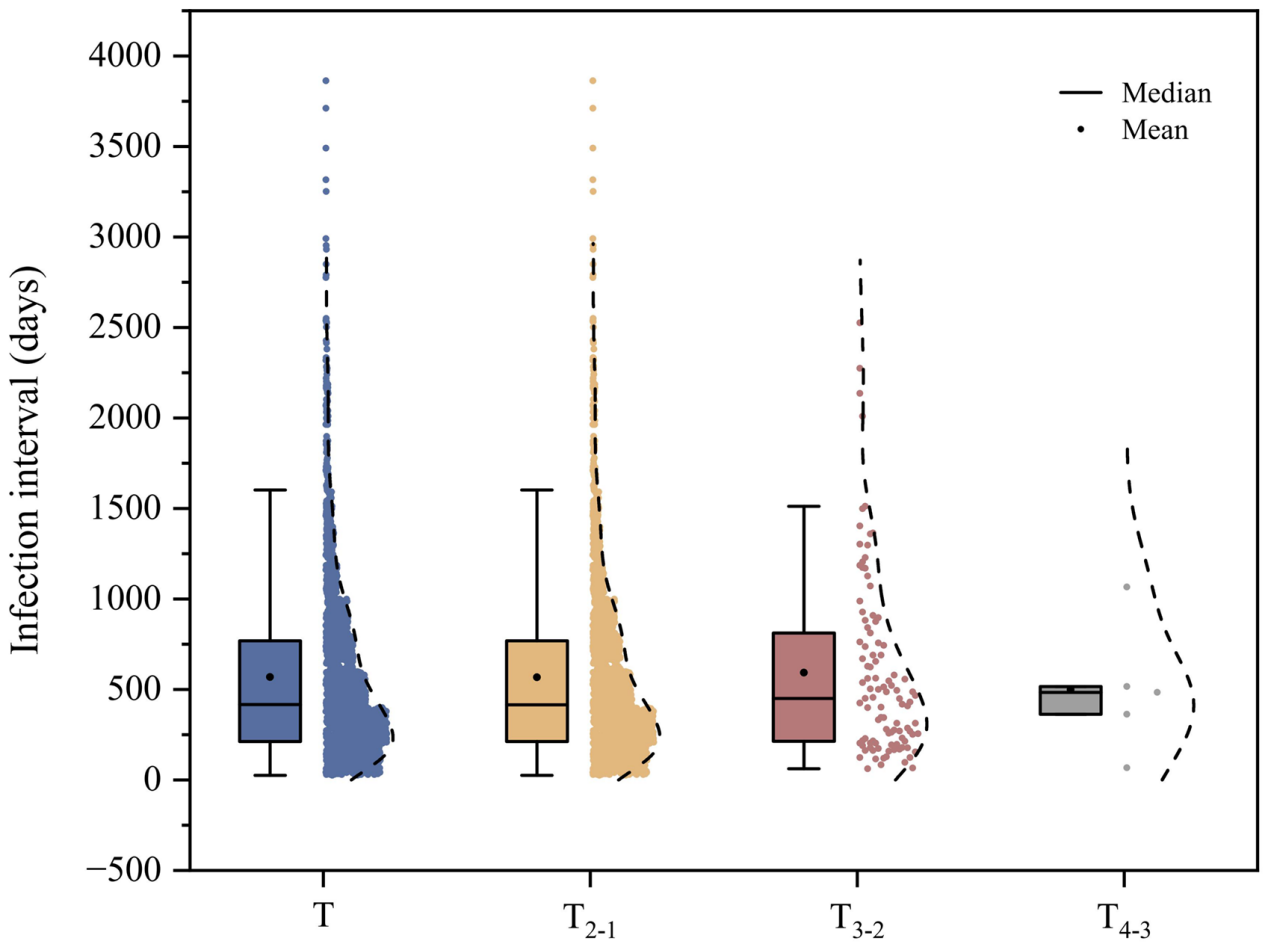
Distribution of infection intervals of reinfections. Note: T is the interval between the onset of all reinfections. T_2-1_ is the interval between the second and first infections. T_3-2_ is the interval between the third infection and the second infection. T_4-3_ is the interval between the fourth and third infections.

The interval between the onset of disease in 2,436 reinfection individuals ranged from 26 to 3,863 days, with a mean of 568.55 days and a median of 416 days. The interval between the second and first infections ranged from 26 to 3,863 days, with a mean value of 567.64 days and a median of 415 days. The time interval between the third and second infections ranged from 62 to 2,526 days, with a mean of 592.45 days and a median of 450 days. The time interval between the fourth and third infections ranged from 67 to 1,066 days, with a mean of 499.20 days and a median of 484 days, as shown in Figure 3.

Reinfection occurred within 1 year in 1,064 (43.68%), within 1–2 years in 678 (27.83%), within 2–3 years in 400 (16.42%) and more than 3 years in 294 (12.07%).

#### 3.1.2 Population distribution

HFMD reinfection occurs as young as 3 months and as old as 14 years. 91.83% occurred in younger children aged 5 years and below, with reinfection rates of 8.61% in children >3 years old and 4.68% in children ≤3 years old. Regarding gender distribution, the reinfection rate was 5.87% in males and 4.93% in females, with a sex ratio of 1.65:1. The population classification was 3.97% for scattered children (children who do not reach the age of kindergarten are cared for by their family members), 7.89% for kindergarten children, and 6.26% for the rest of the population.

#### 3.1.3 Regional distribution

**Figure 4:**
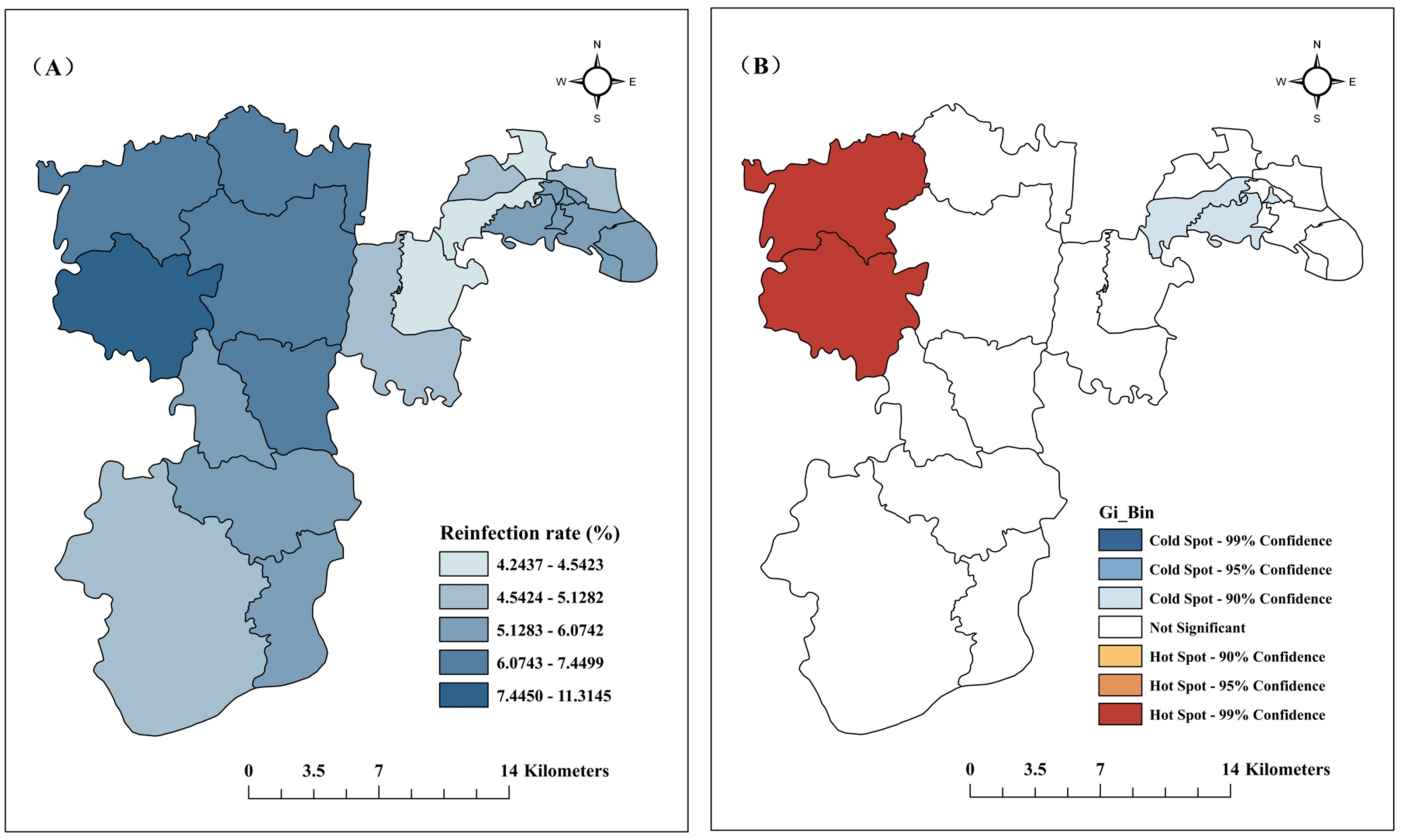
Regional distribution of reinfections in Jiulongpo District, 2009–2023. **(A)** Reinfection rates by town; **(B)** Local spatial autocorrelation analysis of HFMD reinfection.

HFMD reinfectios were reported in all 19 towns from 2009 to 2023. The top 5 towns in terms of reinfection rate were Zouma (11.31%), Shiban (7.45%), Jinfeng (6.69%), Baishiyi (6.61%), Hangu (6.61%), Yuzhoulu had the lowest reinfection rate (4.24%), as shown in Figure 4A. The global spatial autocorrelation Moran’s I coefficient was 0.359, *p* < 0.05, indicating a positive spatial correlation of HFMD reinfection in Jiulongpo District. The results of the local spatial autocorrelation analysis are shown in Figure 4B, with the hot spots concentrated in rural areas and the cold spots concentrated in urban areas.

### 3.2 Analysis of factors influencing HFMD reinfection

The chi-square test results showed that gender, age, population classification, region, and whether it was an epidemic year were associated with HFMD reinfection, and the difference was statistically significant (*p* < 0.05), as shown in Table 3.

**Table 3:** Univariate analysis of HFMD reinfection in Jiulongpo District, 2009–2023.

Variables		Non-reinfection	Reinfection	Reinfection rate (%)	*χ^2^*	*p*
Gender	Male	24,336	1,518	5.87	18.45	<0.001
	Female	17,696	918	4.93		
Age (years)	Age>3	8,279	780	8.61	215.54	<0.001
	Age ≤ 3	33,753	1,656	4.68		
Population classification	Others	1,558	104	6.26	299.43	<0.001
	Kindergarten children	14,855	1,272	7.89		
	Scattered children^*^	25,619	1,060	3.97		
Region	Rural	11,596	726	5.89	5.64	0.018
	Urban	30,436	1,710	5.32		
Clinical type	Mild	41,989	2,434	5.48	0.09	0.760
	Severe	43	2	4.44		
Peak period	No	9,796	556	5.37	0.30	0.584
	Yes	32,236	1,880	5.51		
Epidemic year	No	16,387	1,134	6.47	55.19	<0.001
	Yes	25,645	1,302	4.83		

^*^ Scattered children are defined as children who do not reach the age of kindergarten are cared for by their family members.

As shown in Table 4, multifactorial logistic regression analysis with reinfection as the dependent variable and variables with statistically significant results from the univariate analysis (gender, age, population classification, region, and epidemic year) were used as the independent variables, which showed that males (OR = 1.184, 95% CI: 1.088 ~ 1.288), >3 years old (OR = 1.417, 95% CI: 1.269 ~ 1.582), kindergarten children (OR = 1.524, 95% CI: 1.231 ~ 1.886), rural areas (OR = 1.132, 95% CI: 1.034 ~ 1.238), and non-epidemic year (OR = 1.317, 95% CI: 1.212 ~ 1.430) were the risk factors for reinfection with HFMD (*p* < 0.05).

**Table 4:** Logistic regression analysis of HFMD reinfection in Jiulongpo District, 2009–2023.

Variables		B	S.E.	Wald *χ^2^*	*p*	OR (95% CI)
Intercept		−3.440	0.047	5,343.065	<0.001	0.032
Gender	Female	1				
	Male	0.169	0.043	15.283	<0.001	1.184 (1.088 ~ 1.288)
Age (years)	Age ≤ 3	1				
	Age>3	0.615	0.068	38.223	<0.001	1.417 (1.269 ~ 1.582)
Population classification	Others	1				
	Kindergarten children	0.421	0.109	14.928	<0.001	1.524 (1.231 ~ 1.886)
	Scattered children^*^	−0.136	0.119	1.306	0.253	0.873 (0.691 ~ 1.102)
Region	Urban	1				
	Rural	0.124	0.046	7.251	0.007	1.132 (1.034 ~ 1.238)
Epidemic year	Yes	1				
	No	0.275	0.042	42.769	<0.001	1.317 (1.212 ~ 1.430)

^*^ Scattered children are defined as children who do not reach the age of kindergarten are cared for by their family members.

## 4 Discussion

The reinfection rate of HFMD in Jiulongpo District from 2009 to 2023 was 5.48%, which was higher than the reinfection rates reported in other provinces and cities, such as Guangzhou City (3.07%) (25), Anhui Province (2.02%) (21), and Wuhan City (1.93%) (20), and it was an area to focus on. The number of cases in different regions, the time interval of the study, the criteria for determining repeat cases, and the climatic conditions all impact the study results, thus contributing to the differences in reinfection rates.

In terms of temporal distribution, HFMD reinfections occurred in all months of the year, with a bimodal epidemic pattern in April–July and October–November, which was the same as in Hefei City (26) but differed from the single-peak epidemic pattern (April–July) in Guangzhou City (25). Relative humidity increases the risk of HFMD reinfection (26). Jiulongpo District is located in a foggy basin topography, and the higher humidity in autumn provides favorable conditions for the survival and transmission of enteroviruses; in contrast, Guangzhou has a significant drop in humidity and persistently high temperatures after the summer season, which may inhibit the transmission of viruses in autumn to some extent. The results of the univariate analysis showed that reinfection was not associated with peak or non-peak periods (*p* > 0.05) and that the rate of reinfection during off-peak periods was essentially the same as that during peak periods despite lower incidence, suggesting that the risk of reinfection was the same throughout the year in all periods. The results of the multifactorial analysis showed that the risk of reinfection in non-epidemic years was 1.317 times higher than that in epidemic years (95% CI, 1.212 ~ 1.430, *p*<0.05), indicating that there were more new cases in epidemic years, and more reinfected cases in non-epidemic years, we suggest that the Health Commission should adjust the direction of prevention and control promptly after the epidemic period, with a focus on reducing repeat infections, for example, by targeting prevention and control guidelines to those who have been infected via text message on cell phone. After the first HFMD infection, reinfection would occur in an average of 568.55 days (18.95 months), which was a longer time interval than the data reported in Hainan Province (426.92 days) (27) and Shapingba District of Chongqing (15.7 months) (28); 71.51% of the patients were reinfected within 2 years, the Education Commission regularly conducts publicity on HFMD prevention and control in kindergartens to raise guardians’ awareness of disease prevention.

In terms of regional distribution, there were reinfections in all towns, and the occurrence of reinfection in rural areas was 1.132 times higher than that in urban areas (95% CI, 1.034 ~ 1.238, *p* < 0.05); local spatial autocorrelation analysis presented the same results, with hot spots concentrated in rural areas and cold spots in urban areas. With the increase in the urbanization rate, young and middle-aged people in rural areas gradually move to cities, leaving behind the older adult and infants, who are relatively weak in terms of medical awareness and knowledge of disease prevention (8). At the same time, rural areas themselves have poorer health resources, living conditions, and lower education (25), all of which can cause HFMD reinfection in rural areas. We recommend that the Government strengthen the construction of health infrastructure in rural areas, improve the living environment and sanitary conditions, and provide clean water and sanitation facilities. It should also popularize knowledge of HFMD prevention through publicity posters and radio broadcasts to raise residents’ awareness of prevention and control. The Health Commission should strengthen training and support for primary healthcare organizations in rural areas to enhance their capacity for early diagnosis and treatment of HFMD.

In terms of population distribution, men are at higher risk of reinfection, which can be attributed to the fact that men prefer outdoor activities and have a wider range of activities than women, which increases the chances of exposure (29–31); at the same time, compared with women, men are less alert to the risk of exposure and less adherent to hygiene practices, which increases susceptibility (32). In addition, it has been shown that men and women have different immunological profiles, with a higher detection rate of enteroviruses in men than in women (33). Unlike other studies (34) that suggested higher reinfection rates in children <4 years of age, children >3 years of age in kindergarten were the risk factor in our study; there is evidence (35) that children aged 4–11 years are more susceptible to extreme sunlight exposure than children <4 years of age, resulting in a relatively high proportion of HFMD reinfection in children in childcare. Given the high population density, small activity space, high contact exposure, and high possibility of reinfection in childcare institutions, kindergartens must strictly implement a hygiene and disinfection system to regularly clean and disinfect classrooms, toys, and tableware. Early childhood is a critical period of human development (36), and kindergarten is a critical stage for forming habits. We suggest that kindergartens set hygiene rules with children (e.g., washing hands before and after meals and after outdoor lessons), post these rules in charts and graphs in conspicuous places, and give rewards for complying with the rules to help them form habits.

There are several limitations to this study. First, HFMD was only included in the statutory reporting of infectious diseases in May 2008, so the rate of HFMD reinfection since 2009 may have been underestimated, and the study findings may be subject to some information bias. Secondly, factors affecting HFMD reinfection were not fully considered in this study, and weather and social contact factors will be included in future studies to improve the interpretability of the study. Finally, there were no cases of HFMD in which the laboratory detected both the first infection and reinfection, so it was impossible to determine whether the same virus or a different virus caused the reinfection.

## 5 Conclusion

In summary, HFMD reinfection was more serious in Jiulongpo District from 2009 to 2023. The reinfection risk was higher in non-epidemic years, male, living in rural areas, >3 years old, and kindergarten children. Post-epidemic prevention and control measures should prioritize interventions to target reinfection, focusing on children in rural areas and kindergartens. We recommended that the government should improve the infrastructure and sanitary conditions in rural areas, conduct regular publicity in kindergartens to raise guardians’ awareness of disease prevention, continue to improve the training of primary health care institutions to enhance the ability of early diagnosis of HFMD and help children in childcare to develop good hygiene habits, in the hope of effectively decreasing the reinfection rate of HFMD. As a next step, we will focus on the reasons for the higher risk of reinfection in non-endemic years and explore whether alternating viral serotypes are related.

## Data Availability

The original contributions presented in the study are included in the article/supplementary material, further inquiries can be directed to the corresponding author.
