# Corrigendum: Epidemiological characteristics and influencing factors of hand, foot and mouth disease reinfection cases in Jiulongpo District, Chongqing, China, 2009-2023

**DOI:** 10.3389/fpubh.2025.1601896

**Published:** 2025-04-10

**Authors:** Huixian Zhou, Yuan Yao, Qianjin Long, Chunyan Deng

**Affiliations:** Center for Disease Control and Prevention of Jiulongpo District, Chongqing, China

**Keywords:** hand, foot and mouth disease, reinfection, epidemiological characteristics, spatial autocorrelation, influencing factors

In the published article, there was an error. A correction has been made to **Introduction**, Paragraph 3. This sentence previously stated:

“From 2008 to 2015, 398,010 out of 820,000 HFMD patients in China were diagnosed with reinfection cases”

The corrected sentence appears below:

“From 2008 to 2015, 398,010 HFMD patients (a total of ≈ 820,000 episodes) in China were diagnosed with reinfection cases”

The authors apologize for this error and state that this does not change the scientific conclusions of the article in any way. The original article has been updated.

